# Serum Lipoprotein Profile Is Associated With Protective Effects of Oral Contraceptive Use on Multiple Sclerosis Severity: A Cross-Sectional Study

**DOI:** 10.3389/fneur.2019.00060

**Published:** 2019-02-05

**Authors:** Armando Sena, Ana Macedo, Véronique Ferret-Sena, Carlos Capela, Rui Pedrosa

**Affiliations:** ^1^Centro de Investigação Multidisciplinar Egas Moniz (CiiEM), Instituto Universitário Egas Moniz, Caparica, Portugal; ^2^Faculdade de Ciências Médicas, Universidade Nova de Lisboa, Lisbon, Portugal; ^3^Departamento de Neurociências, Serviço de Neurologia, Centro Hospitalar de Lisboa-Central, Lisbon, Portugal; ^4^Keypoint, Consultora Científica, Algés, Portugal; ^5^Departamento de Ciências Biomédicas e Medicina (DCBM), Universidade do Algarve, Faro, Portugal

**Keywords:** multiple sclerosis, lipoproteins, cholesterol, apolipoprotein E, oral contraceptives, sex steroids

## Abstract

**Background:** The mechanisms underlying the influence of sex hormones in multiple sclerosis (MS) are uncertain. Sex steroids interact with cholesterol metabolism and the serum lipid profile has been associated with the severity of the disease. We hypothesized that the putative associations between lipoprotein metabolism and MS could be modulated by sex steroids exposure. The aim of this study was to investigate whether oral contraceptives (OC) use changes the lipoprotein profile associated with disability in patients with multiple sclerosis.

**Methods:** Clinical data was collected from 133 relapsing-remitting multiple sclerosis (RRMS) women with a mean of 6.5 years of disease duration and prior to the start of disease-modifying therapies. Patients who were using OC after disease onset (DO) (OC+, *n* = 57) were compared to those who never used OC or discontinued its intake before DO (OC–, *n* = 76). In both cohorts of subjects, the associations between the apolipoprotein E (ApoE) polymorphism, and plasma lipid levels, and the annualized relapse rate (RR), the Expanded Disability Status Score (EDSS), and the Multiple Sclerosis Severity Score (MSSS) were evaluated using a hierarchic multiple regression analysis after adjustment for confounders.

**Results:** Low density lipoprotein (LDL) levels were associated with higher EDSS (*p* = 0.010) and MSSS (*p* = 0.024) in the whole studied cohort. In E3/E3 phenotype carriers (73.7%), EDSS and MSSS were lower in OC+ in comparison with OC– subgroup of patients (*p* < 0.01). LDL and total cholesterol were associated with EDSS (*p* = 0.005 and *p* = 0.043, respectively), and LDL and the triglyceride/high density lipoprotein ratio with MSSS (*p* = 0.011 and *p* = 0.048, respectively) in OC+ patients. In OC– subgroup of patients, ApoE levels were associated with EDSS (*p* = 0.012) and MSSS (*p* = 0.031). No significant interactions between the lipid variables or OC use and RR were observed.

**Conclusions:** Serum lipid profile is associated with protective effects of OC use on disability of RRMS patients. Lipoprotein metabolism may be involved in the modulatory effects of sex steroids on the severity of the disease.

## Introduction

Multiple sclerosis (MS) is an inflammatory demyelinating and neurodegenerative disease in which onset and course may be modulated by gender and sex hormones (1). The mechanisms underlying sex differences and effects of sex steroids in the disease are poorly understood. Recent studies have implicated serum cholesterol metabolism and lipoprotein profile in the pathophysiology and severity of the disease (2). Nevertheless, results are mixed, and whether these relations are causal or secondary to the disease process, treatment regimens or other confounding factors remain uncertain. Complex interactions between cholesterol metabolism and sex steroids are well-known (3) and may modulate the clinical activity of experimental autoimmune encephalomyelitis (EAE), the animal model for MS (4, 5). Therefore, we hypothesized that the putative associations between lipoprotein metabolism and the severity of MS could be modulated by sex hormone exposure.

Oral contraceptives (OC) use has provided an opportunity to assess the influence of sex hormones on the risk and course of MS. Recent data suggested a less severe clinical course (6, 7) and decreased inflammatory brain lesions (8) in relapsing-remitting (RRMS) women taking OC. Protective effects of estriol, the estrogen unique to parity (9), and of hormone therapy in postmenopausal MS women were also reported (10). In healthy women, OC use induces variable alterations in serum lipids and apolipoproteins levels modulated by ApoE polymorphism (11, 12). Although ApoE polymorphism is not generally considered to affect the risk of MS, its association with the neurodegenerative process and severity of the disease is still controversial (4, 5). ApoE is implicated in the immune dysfunction and clinical activity of EAE (5) and recent studies have suggested that EAE disease severity is differently modulated by cholesterol and ApoE metabolism in female and male mice (4, 13). Oestrogens may regulate the expression of ApoE gene (2) and estrogen treatment has protective effects in EAE (14). Oestrogens-ApoE interactions are suggested to be involved in other neurological conditions with a sex bias and abnormal cholesterol metabolism, such as Alzheimer disease (15). Based on these data, the aim of the present study was to investigate whether OC intake in RRMS patients influence the associations between the serum lipoprotein profile and the clinical severity of the disease.

## Materials and Methods

### Study Population

The studied population include 133 women with the diagnosis of RRMS according to the revised McDonald criteria (16) followed at the MS outpatient clinic of a University Hospital in Lisbon (Portugal). Most women enrolled in this study belong to a population of Caucasian origin included in a previous work published by our group (7). The present study includes all patients followed in our clinic since 1995 diagnosed with RRMS with at least 2 years of disease duration and whose lipid data were available prior to the start of disease-modifying therapies. No woman was taking lipid-lowering agents. Disease onset (DO) was defined as the age of appearance of the first symptoms suggestive of MS. The annualized relapse rate (RR) and the Expanded Disability Status Scale (EDSS) and Multiple Sclerosis Severity (MSSS) values were determined at a stable phase of the disease. MSSS scores were obtained from Figure 3 of the paper of Roxburg et al. (17). The MSSS is based on EDSS scores adjusted for disease duration and it is a method to compare disability progression in groups of patients (7, 17). Women in menopause or history of gynecological surgical interventions or a delivery the last 6 months were excluded. Clinical indexed information included the body mass index (BMI) (Kg/m^2^), age of first menstruation (menarche) and history of childbirths (parity), smoking habit, and OC intake. Patients labeled smokers reported to smoke regularly at least five cigarettes per day since DO. Women were classified as OC non-users if they never used OC or discontinued its intake for at least 1 year before DO (OC–); and OC users if they maintained pill intake after DO for at least a continuous period of 1 year (OC+). We were unable to take the composition of the prescribed pill into account because this information was lacking for some patients and many changed the brand of the drug. However, all women who remembered the formulation of OC used took formulations of 20 or 30 μg of ethinyl estradiol combined with progestin. No woman reported to use progestin-only formulations. This study was approved by the Ethics Committee of the Centro Hospitalar, Lisboa Central (Lisbon, Portugal). All patients gave written informed consent, including for publication of results, in accordance with the Declaration of Helsinki.

### Biochemical Analysis

Blood samples were collected in fasting conditions shortly after clinical data collection and neurological examination. Plasma or serum samples were stored at −80°C and biochemical measurements performed in blind conditions regarding subject participants. At sampling, patients were in a remission phase of RRMS and none of them had initiated disease-modifying therapies, suffered from a relapse or were treated with steroids for at least 1 month. Serum triglycerides (TG), total cholesterol (TC), high density lipoprotein (HDL)-cholesterol, apolipoprotein A-1 (ApoA1), and apolipoprotein B (ApoB) were determined with enzymatic methods and lipoprotein (a) [Lp(a)] by turbidimetric immunoassay by using a Hitachi 911 autoanalyzer and commercial kits (Roche Diagnostic, Mannheim, Germany). Non-HDL cholesterol levels were calculated by subtracting HDL from TC. Low density lipoprotein (LDL)-cholesterol was determined by using the Friedewald equation (18) and oxidized LDL (oxLDL) by Enzyme-Linked Immunosorbent Assay (Mercodia, eBioscience). Apolipoprotein E (ApoE) protein levels were determined by electroimmunodiffusion (Sebia, Emery, France) and ApoE polymorphism examined by using an isoelectric focusing (IEF) method as described previously (19). Briefly, 15 μl of delipidated plasma samples was run on agarose with sorbitol, urea, ampholine (pH 5–7) and pharmalyte (pH 4–6.5) (Amersham Pharmacia Biotech, Little Chalfont, UK). After IEF, the proteins were transferred to nitrocellulose membranes (Immobilon, pore size 0.2 μm; Millipore Corporate Headquarters, Billerica, USA). The membranes were incubated with polyclonal-goat anti-human ApoE antibody (Daichi Pure Chemicals, Tokyo, Japan) and IgG peroxidase-conjugate anti-goat antibody (Sigma-Aldrich Biotechnology, St Louis, USA). The ApoE isoforms were visualized in a solution containing 3,3' diaminobenzidine tetrahydrochloride reagent (Sigma). For common ApoE polymorphism, protein phenotyping is in good agreement with DNA-based genotyping (20).

### Statistical Analysis

Patient demographic and clinical characteristics were described using mean, median, standard deviation, and interquartile range for continuous variables. In the text, standard deviation is presented as mean (standard deviation). For categorical variables absolute and relative frequencies were calculated. The relation between severity parameters of clinical disease activity (RR, MSSS, and EDSS) and ApoE phenotypes was evaluated using Kruskal-Wallis ranking test. Comparisons of disease severity between OC+ and OC– subgroups of patients were performed using a Mann Whitney test. The associations between disease severity parameters such as MSSS, EDSS, and RR and the lipid profile were evaluated using a two tailed Spearman Correlation analysis. The correlation analysis was performed for the total subset of patients carrying the E3/E3 phenotype and also split for those in OC+ and OC– subgroups. A hierarchical multiple linear regression was used assuming the EDSS and MSSS as dependent variables and characterization variables such as age, disease onset, oral contraception, disease duration and parity (block 1), and lipid profile parameters that had significant correlation with EDSS and MSSS in the correlation analysis (block 2), as independent variables. A enter model was used for the variables in the block 1 and a stepwise model was used for variables in block 2.A significance level of 0.05 was considered in all analysis.

## Results

The main demographic and clinical characteristics of the studied population are summarized in Table 1. Twenty-nine patients (21.8%) were classified as overweight (25 ≥ BMI < 30) or obese (BMI ≥ 30). In the OC+ subgroup (*n* = 57), the mean duration of OC use was 10 years (6.6) and all but nine women started intake before DO. In the OC– subgroup, fifty patients were never prescribed with OC and 26 discontinued the intake before DO. OC+ patients were younger and had the onset of the disease at an earlier age than OC– subgroup of patients. Significant associations were found between EDSS and MSSS and age (*p* < 0.001 and *p* = 0.013), DO (*p* = 0.004 and *p* < 0.001), disease duration (*p* = 0.004 and *p* < 0.001), OC use (*p* = 0.001 and *p* = 0.002), and parity after DO (*p* < 0.001 and *p* = 0.006). RR was only associated with disease duration (*p* = 0.006). Menarche age, duration of OC intake, BMI, and smoker habit were not associated with RR or disability scores (data not shown). Concerning the lipid data, HDL and Apo A1 levels were higher in OC+ patients. In a hierarchic multiple regression analysis adjusted for age, DO, disease duration, and OC use, LDL was the only lipid variable associated with EDSS and MSSS (β = 0.008, 95% CI (0.002 to 0.015) *p* = 0.010 and β = 0.013, 95% CI (0.002 to 0.025) *p* = 0.024, respectively). ApoE phenotypes found in the studied cohort were E3/E3 (*n* = 98, 73.7%), E4/E3 (*n* = 20, 15%), E2/E3 (*n* = 12, 9%). Analysis of ApoE polymorphism was missing from one patient and two additional patients carried the E4/E2 phenotype. No homozygotes for the E4 and E2 alleles were detected. The observed frequencies of ApoE alleles were comparable with those reported for the general populations in Portugal and other countries in South Europe (21).

**Table 1:** Patient demographic and clinical characteristics.

**Characteristics**	**Total (*n* = 133)**	**OC+** **(*n* = 57)**	**OC–** **(*n* = 76)**	***p-value***
Age (years)	35.2 ± 8.4	32.4 ± 6.9	37.3 ± 8.8	**0.001**
Disease onset (age)	28.6 ± 8.2	25.7 ± 6.7	30.8 ± 8.5	** <0.001**
Disease duration (years)	6.5 ± 5.3	6.6 ± 5.0	6.4 ± 5.5	0.836
EDSS	2.1 ± 1.3 2.0 [1.0;3.0]^$^	1.7 ± 1.1 1.5 [1.0;2.0]^$^	2.4 ± 1.4 2.5 [1.6;3.5]^$^	** <0.001**
MSSS	3.5 ± 2.4 3.4 [1.5;5.2]^$^	2.8 ± 2.0 2.3 [1.3;4.5]^$^	4.1 ± 2.6 3.9 [2.0;6.0]^$^	**0.003**
Relapse rate	0.9 ± 0.6 1.0 [0.5;1.0]^$^	0.9 ± 0.5 1.0 [0.5;1.0]^$^	± 0.6 1.0 [0.5;1.0]^$^	0.317
TC (mg/dl)	201.5 ± 36.5	202.9 ± 34.7	200.5 ± 37.9	0.708
LDL (mg/dl)	126.0 ± 34.1	123.2 ± 33.1	128.1 ± 34.9	0.418
HDL (mg/dl)	57.9 ± 15.2	60.9 ± 17.8	55.7 ± 12.5	**0.049**
Non HDL (mg/dl)	143.6 ± 37.4	142.0 ± 35.8	144.8 ± 38.9	0.670
Oxidized LDL (u/L)	60.3 ± 24.3	61.0 ± 25.3	59.4 ± 23.9	0.863
TG (mg/dl)	95.3 ± 47.6	102.2 ± 49.5	90.1 ± 45.8	0.149
ApoA1 (mg/dl)	159.1 ± 33.7	173.0 ± 37.6	148.5 ± 26.1	** <0.001**
ApoB (mg/dl)	90.2 ± 24.7	91.2 ± 22.9	89.5 ± 26.1	0.698
Lp(a) (mg/dl)	29.4 ± 29.5	32.4 ± 29.5	27.5 ± 29.6	0.377
ApoE (mg/l)	77.2 ± 31.8	70.4 ± 29.1	81.9 ± 32.9	0.052
ApoE 3/3 n (%)	98 (73.7)	42 (73.7)	56 (73.7)	0.184
ApoE 4/3 n (%)	20 (15.0)	12 (21.1)	8 (10.5)	
ApoE 2/3 n (%)	12 (9.0)	3 (5.3)	9 (11.8)	

*The continues variables are expressed as mean ± SD*;

$ * median and IQR []*.

*Categorical variables are expressed as frequency n (%). p represents the significance of the comparison between OC user (OC+) and non-user (OC–) patients (Mann–Whitney test). EDSS, Expanded Disability Status Scale; MSSS, Multiple Sclerosis Severity Score; TC, total cholesterol; LDL, low density lipoprotein; HDL, high density lipoprotein TG, triglyceride; Apo A1, apolipoprotein A1; ApoB, apolipoprotein B; Lp(a), lipoprotein (a) ApoE, apolipoprotein E; ApoE phenotypes (3/3, 4/3,2/3). Analysis of ApoE polymorphism was missing from one patient and two additional patients carried the E4/E2 phenotype (not shown). Bold values mean significant differences*.

**Figure 1:**
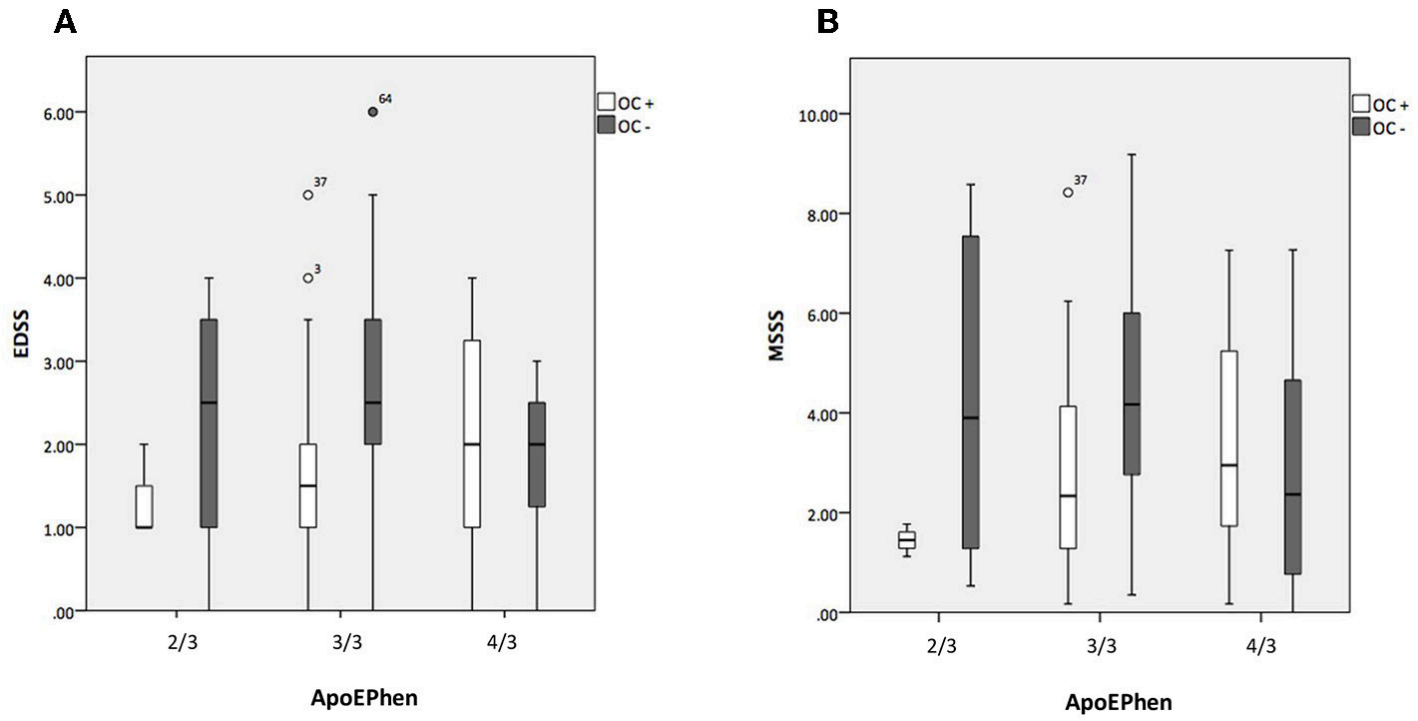
Association between Apolipoprotein E phenotypes and disability changes stratified according to oral contraceptive use. Dependence of the Expanded Disability Status Scale (EDSS) **(A)** and of the Multiple Sclerosis Severity Score (MSSS) **(B)** with Apo E phenotypes (ApoEPhen) (2/3, 3/3, 4/3) in oral contraceptive users (*n* = 57 in white) and non-users (*n* = 73 in gray) subgroups of patients. The bars represent inter-quartile range (percentiles 25 and 75); OC+, ApoEPhen 2/3- only 3 patients analyzed. EDSS and MSSS values are lower in OC users in comparison to non-user patients carrying the E3/E3 phenotype (Mann-Whitney test, *p* < 0.001 and *p* = 0.001, respectively).

No associations between the three common ApoE phenotypes and EDSS, MSSS, or RR were found. However, in the E3/E3 subset of subjects, EDSS, and MSSS values were lower in OC+ in comparison to OC– subgroup of patients (*p* < 0.01) (Figure 1). These results remain significant after hierarchical multiple linear regression analysis adjusting for demographic features among the ApoE genetic groups. RR was not significantly changed by OC intake (*p* = 0.457). In consequence, serum lipid and apolipoprotein levels were investigated in this subset of patients according to OC use. Overall, there was no statistical difference in the lipid profile with the exception of higher ApoA1 and lower ApoE levels in OC+ in comparison to OC– patients [171.3 mg/dl (40.6) vs. 151.5 mg/dl, (27.5); *p* < 0.01 and 67.3 mg/dl (29.6) vs. 80.1 mg/dl (28.7); *p* < 0.05, respectively]. Correlation between lipoprotein levels and disability scores were analyzed in E3/E3 subset of patients stratified according to OC use. In OC+ subgroup of patients, LDL was associated with EDSS (*p* = 0.018) and ApoB was associated with MSSS (*p* = 0.043). In contrast, in the OC– subgroup of patients, ApoE was associated with MSSS values (*p* = 0.008); TC and non-HDL were associated with EDSS (*p* = 0.025 and *p* = 0.035, respectively); and TC (*p* = 0.035), LDL (*p* = 0.028), non-HDL (*p* = 0.005), and ApoB (*p* = 0.008) with RR (Table 2).

**Table 2:** Associations between the lipid profile and clinical variables in patients carrying the E3/E3 phenotype stratified according to oral contraceptive use.

**Lipid variables**	**OC+**			**OC–**		
	**RR**	**EDSS**	**MSSS**	**RR**	**EDSS**	**MSSS**
TC (mg/dl)	−0.034 (0.833)	0.211 (0.180)	0.186 (0.239)	**0.283** **(0.035)**	**0.299** **(0.025)**	0.203 (0.134)
LDL (mg/dl)	−0.017 (0.917)	**0.363** **(0.018)**	0.255 (0.103)	**0.294** **(0.028)**	0.246 (0.067)	0.128 (0.346)
Non-HDL (mg/dl)	−0.045 (0.778)	0.268 (0.086)	0.152 (0.338)	**0.366** **(0.005)**	**0.283** **(0.035)**	0.221 (0.101)
Apo B (mg/dl)	0.103 (0.517)	0.270 (0.084)	**0.313** **(0.043)**	**0.357** **(0.008)**	0.230 (0.094)	0.199 (0.150)
Apo E (mg/l)	−0.001 (0.995)	−0.002 (0.993)	0.030 (0.863)	0.154 (0.266)	0.206 (0.136)	**0.356** **(0.008)**

*The results show Spearman Correlation significance between subgroups of patients carrying the E3/E3 phenotype. OC+: oral contraceptives user (n = 36); OC– oral contraceptives non-user (n = 51); RR, annualized relapse Rate; EDSS, Expanded Disability Status Scale; MSSS, Multiple Sclerosis Severity Score; TC, total cholesterol; LDL, low density lipoprotein; HDL, high density lipoprotein; ApoB, apolipoprotein B; ApoE, apolipoprotein E. No significant associations were found for other lipid variables (not shown). Bold values mean significant differences*.

A hierarchic multiple regression analysis in E3/E3 subjects split for OC+ and OC– patients and adjusted for age, DO and disease duration was then performed (Table 3). EDSS was related to LDL [β = 0.026, 95% CI (0.009 to 0.044); *p* = 0.005] and TC [β = −0.018, 95% CI (−0.034 to −0.001); *p* = 0.043]; model *R* square = 0.328 (*p* = 0.015) in OC+ population; and to ApoE [β = 0.015, 95% CI (0.004 to 0.028); *p* = 0.012]; model *R* square = 0.312 (*p* = 0.001) in the OC– subgroup of patients. In a similar model, MSSS was related to LDL, [β = 0.022, 95% CI (0.002 to 0.033); *p* = 0.011] and to TG/HDL ratio, [β = 0.389, 95% CI (0.088 to 0.800); *p* = 0.048]; model *R* square = 0.333 (*p* = 0.016) in OC+ patients; and to ApoE, [β = 0.024, 95% CI (0.003 to 0.046); *p* = 0.031]; model *R* square = 0.299 (*p* = 0.002) in OC– population (see Supplementary Material). No significant associations between the lipids variables and RR were observed using this model (not shown).

**Table 3:** Hierarchical multiple linear regression model to determine the association between lipid variables and disability changes in patients carrying the E3/E3 phenotype stratified according to oral contraceptive use.

**Factors**	β coefficient^*^	CI 95%^*^	***p-value***	***R*** **square**	**Sig** **(ANOVA)**
**DEPENDENT VARIABLE: EDSS**					
**OC+**					
TC (mg/dl)	**−0.018**	−0.034 to −0.001	**0.043**	0.328	**0.015**
LDL (mg/dl)	**0.026**	0.009 to 0.044	**0.005**		
**OC–**					
ApoE (mg/l)	**0.015**	0.004 to 0.028	**0.012**	0.312	**0.001**
**DEPENDENT VARIABLE: MSSS**					
**OC+**					
LDL (mg/dl)	**0.022**	0.002 to 0.033	**0.011**	0.333	**0.016**
TG/HDL ratio	**0.389**	0.088 to 0.800	**0.048**		
**OC–**					
ApoE (mg/l)	**0.024**	0.003 to 0.46	**0.031**	0.299	**0.002**

* *Adjusted for age, disease onset, disease duration, and oral contraception (OC) intake. EDSS, Expanded Disability Status Scale; MSSS, Multiple Sclerosis Severity Score; Oral contraceptive users (OC+, n = 34) and non-users (OC–, n = 51) subgroups of patients carrying the E3/E3 phenotype; TC, total cholesterol; LDL, low-density lipoprotein; ApoE, apolipoprotein E; TG, triglycerides; HDL, high-density lipoprotein. No significant associations were found for other lipid variables (not shown). Bold values mean significant differences*.

## Discussion

The results reported in this study suggest that oral OC use modifies the serum lipoprotein profile associated with disability in patients with MS. Recent prospective studies have shown variably associations between serum lipid and apolipoprotein levels and the risk of new lesions accumulation and disability progression in patients with RRMS and/or the first symptoms suggestive of the disease (clinical isolated syndrome, CIS) (2). However, most research has included patients under immunomodulatory therapies and have not assessed possible influences of OC use. In consequence, in this cohort, lipid data was analyzed before the introduction of disease-modifying therapies and comparing women who never used OC or stopped its intake before disease onset (OC–) to those who were OC users after disease onset (OC+).

The serum lipoprotein profile is in part genetically regulated by the common human isoforms of ApoE designated E2, E3, and E4, which display different modulatory roles in cholesterol metabolism, immune function, and neuronal homeostasis (3, 5, 11). In agreement with most studies (4, 5), no association between the ApoE polymorphism and the clinical activity and severity of MS was found. However, in individuals carrying the major E3/E3 phenotype, EDSS and MSSS were significant lower in the OC+ group, when compared to OC– patients. In line with previous retrospective work (6, 7) and a recent longitudinal study (22) no influence of OC use on relapse risk was observed. Future research in a larger population of carriers of the ε2 and ε4 alleles is needed. In particular, the apparent lack of effect on disability in E4/E3 patients is of considerable interest. In fact, experimental and clinical studies have shown that the neuroprotective and anti-inflammatory effects of estrogen are attenuated by the ApoE4 isoform (3) and the risk conferred by this allele for Alzheimer disease is amplified in women (15). These results lead us to perform an analyse of serum lipid variables and their associations with clinical parameters restricted to carriers of the E3/E3 phenotype.

In healthy women, the use of OC formulations containing combinations of ethinyl estradiol and a progestin induce in general an increase of serum TG, ApoA1, and ApoB(11, 12). Higher levels of HDL and/or of its major apolipoprotein, ApoA1, were suggested to be protective for the genesis of new lesions in the MS (23, 24). Although ApoA1 levels were higher in OC+ than in OC– patients, no evidence for a protective effect was observed in agreement with other studies (25–27). In accordance with most studies (27), no independent association between lipid parameters and RR were observed after adjustments in multivariable analysis. In contrast, when all the variables were analyzed in a hierarchic model, significant associations were found between disability and TC, LDL, and the TG/HDL ratio only in the OC+ population. The TG/HDL ratio is a parameter recently associated with insulin resistance, obesity, metabolic syndrome, and clinical outcome in stroke (28). Our finding is consistent with some studies reporting worsening disability in patients with high TG levels (24, 26).

In healthy women, OC intake consistently decreases ApoE levels and changes the distribution of this protein between lipoprotein fractions containing ApoB (LDL and triglyceride-rich lipoproteins) and those devoid of ApoB and rich in ApoA1 (HDL). Interestingly, these alterations are not induced in E4 carriers (12). Recently, differences in LDL particle size were observed between male and female RRMS patients, supporting gender differences in lipid metabolism (29). These data indicate that further work is needed to analyse whether OC intake in these patients modify ApoE distribution among lipid fractions. Nevertheless, ApoE levels were lower in OC+ than in OC– patients, and were correlated in these latter subjects with disability. Previous studies have linked higher plasma ApoE levels with severity of EAE (5), higher disability in RRMS (26) and deep gray matter atrophy in CIS patients (25). Several experimental studies have shown that oestrogens may modulate the interactions between Apo E gene expression and LDL metabolism (3, 13). In this context, it is of great interest that an altered gene expression for ApoE and other proteins implicated in cholesterol synthesis and transport occurs during the development and resolution of CNS lesions in EAE and MS patients (30, 31). In addition, Mailleux et al. (13) have shown that LDL receptor deficiency reduces EAE disease severity in female, but not in male rats, through the induction of ApoE release by macrophages. In line with the reviewed data, the present results strongly support a role of sex steroids in modulating ApoE and related cholesterol metabolism in MS patients.

Beyond the relative small dimension of the cohort, absence of a healthy control population and its cross-sectional design, this study has several other limitations. Considering the models statistical assumptions and the nature of the included clinical variables, the results should be interpreted carefully. Prospective studies are necessary to substantiate a causal role of the lipid profile associated with OC behavior in disability progression. It should be noted that many patients are at present medicated following a first clinical episode and paraclinical evidence suggestive of MS (CIS). Therefore, it is increasingly impractical or unethical to carry out a study on a larger population of patients with the diagnosis of RRMS without taking disease-modifying therapies, which may variably interfere with lipid metabolism (32, 33). Although a healthy control population has not be analyzed, as discussed above, our results suggest that OC intake in these patients and healthy women might interact with similar pathways of lipid metabolism. Further work is warranted to investigate this interesting issue. We were unable to include neuroimaging information, analysis of vitamin D, and inflammatory markers. In particular, vitamin D levels could affect the serum lipid profile in MS patients (34) and the mutual metabolic relationships between oestrogens and vitamin D may be relevant for the pathogenesis of the disease (1). However, in a previous work, we have found no evidence for significant alterations of serum 25-hydroxyvitamin D levels associated with OC use in these patients (7). Information concerning the intake of vitamin D supplements was not available. Nonetheless, the population included in this study was analyzed before the intake of these supplements became a common practice by these patients. Dietary and physical activities were not controlled and the impact of different contraceptive formulations could not be evaluated. Gava et al. (6) did not find any differences in the protective effects of OC use in the clinical course of MS depending on the dose of ethinyl estradiol or the type of progestin. However, the anti-inflammatory effects of oestrogens are dose-dependent (8) and future randomized, double-blind, controlled studies are needed to investigate this issue. The progestin content of these formulations could change the lipid profile (11, 12) and were suggested to affect the risk for MS (35). In conclusion, despite these limitations, our results report new findings, supporting a role of the serum lipid profile in mediating modulatory effects of sex steroids in the severity of MS. In addition, they indicate that further work assessing the effects of specific OC doses and formulations in lipoprotein metabolism of these patients may provide new therapeutic strategies for the disease.

## Author Contributions

AS, CC, and RP contributed to the study design and data collection. AM performed the data analysis. AS wrote the manuscript, which was critically reviewed, and drafted by VF-S. All authors contributed to data interpretation and approved the final version of the manuscript.

### Conflict of Interest Statement

The authors declare that the research was conducted in the absence of any commercial or financial relationships that could be construed as a potential conflict of interest.
