# Supplementary material for: Serum Lipoprotein Profile Is Associated With Protective Effects of Oral Contraceptive Use on Multiple Sclerosis Severity: A Cross-Sectional Study
Source: Front Neurol. 2019 Feb 5;10:60. doi: 10.3389/fneur.2019.00060 (PMC6373519; doi:10.3389/fneur.2019.00060)
Supplement: Supplementary file 1 [file Data_Sheet_1.docx]

**Supplementary material**

Hierarchical multiple linear regression model to determine the association between lipid variables and disability changes in patients carrying the E3/E3 phenotype stratified according to oral contraceptive use – model data

EDSS model data

OC+

| **Model Summary^a,c^** | | | | | | | | | | |
| --- | --- | --- | --- | --- | --- | --- | --- | --- | --- | --- |
| Model | R | R Square | Adjusted R Square | Std. Error of the Estimate | Change Statistics | | | | | Durbin-Watson |
|  |  |  |  |  | R Square Change | F Change | df1 | df2 | Sig. F Change |  |
|  | .573^b^ | .328 | .238 | .97040 | .100 | 4.480 | 1 | 30 | .043 | 1.744 |
| a. OC+ | | | | | | | | | | |
| b. Predictors: (Constant), D_DURAT, dis-ons, LDLc, ColT | | | | | | | | | | |
| c. Dependent Variable: EDSS | | | | | | | | | | |

| **Coefficients^a,b^** | | | | | | | | | | | |
| --- | --- | --- | --- | --- | --- | --- | --- | --- | --- | --- | --- |
| Model | | Unstandardized Coefficients | | Standardized Coefficients | t | Sig. | Correlations | | | Collinearity Statistics | |
|  |  | B | Std. Error | Beta |  |  | Zero-order | Partial | Part | Tolerance | VIF |
|  | (Constant) | 1.572 | 1.246 |  | 1.262 | .217 |  |  |  |  |  |
|  | dis-ons | .007 | .030 | .038 | .243 | .810 | -.038 | .044 | .036 | .910 | 1.099 |
|  | D_DURAT | .052 | .039 | .216 | 1.348 | .188 | .317 | .239 | .202 | .869 | 1.151 |
|  | LDLc | .026 | .009 | .941 | 2.994 | .005 | .406 | .480 | .448 | .227 | 4.409 |
|  | ColT | -.018 | .008 | -.657 | -2.117 | .043 | .184 | -.360 | -.317 | .232 | 4.307 |
| a. OC+ | | | | | | | | | | | |
| b. Dependent Variable: EDSS | | | | | | | | | | | |

| **Residuals Statistics** |
| --- |

|  | Minimum | Maximum | Mean | Std. Deviation |
| --- | --- | --- | --- | --- |
| Predicted Value | .6416 | 2.7380 | 1.6809 | .61216 |
| Residual | -2.09640 | 2.63853 | -.07380 | .96112 |
| Std. Predicted Value | -1.639 | 1.652 | -.007 | .961 |
| Std. Residual | -2.160 | 2.719 | -.076 | .990 |

OC –

| **Model Summary^a,c^** | | | | | | | | | | |
| --- | --- | --- | --- | --- | --- | --- | --- | --- | --- | --- |
| Model | R | R Square | Adjusted R Square | Std. Error of the Estimate | Change Statistics | | | | | Durbin-Watson |
|  |  |  |  |  | R Square Change | F Change | df1 | df2 | Sig. F Change |  |
|  | .605^b^ | .366 | .312 | 1.13908 | .092 | 6.852 | 1 | 47 | .012 | 1.295 |
| a. OC- | | | | | | | | | | |
| b. Predictors: (Constant), D_DURAT, dis-ons, AGE, ApoE | | | | | | | | | | |
| c. Dependent Variable: EDSS | | | | | | | | | | |

| **Coefficients^a,b^** | | | | | | | | | | | |
| --- | --- | --- | --- | --- | --- | --- | --- | --- | --- | --- | --- |
| Model | | Unstandardized Coefficients | | Standardized Coefficients | t | Sig. | Correlations | | | Collinearity Statistics | |
|  |  | B | Std. Error | Beta |  |  | Zero-order | Partial | Part | Tolerance | VIF |
|  | (Constant) | -1.118 | .833 |  | -1.342 | .186 |  |  |  |  |  |
|  | AGE | .167 | .118 | 1.047 | 1.419 | .162 | .484 | .203 | .165 | .025 | 40.334 |
|  | dis-ons | -.116 | .115 | -.675 | -1.007 | .319 | .226 | -.145 | -.117 | .030 | 33.331 |
|  | D_DURAT | -.039 | .122 | -.164 | -.323 | .748 | .387 | -.047 | -.038 | .052 | 19.112 |
|  | ApoE | .015 | .006 | .326 | 2.618 | .012 | .232 | .357 | .304 | .867 | 1.153 |
| a. OC- | | | | | | | | | | | |
| b. Dependent Variable: EDSS | | | | | | | | | | | |

| **Residuals Statistics** |
| --- |

|  | Minimum | Maximum | Mean | Std. Deviation |
| --- | --- | --- | --- | --- |
| Predicted Value | .8526 | 4.4452 | 2.5599 | .82426 |
| Residual | -2.55261 | 2.64024 | -.02287 | 1.09453 |
| Std. Predicted Value | -2.075 | 2.249 | -.020 | .992 |
| Std. Residual | -2.241 | 2.318 | -.020 | .961 |

Model MSSS

OC+

| **Model Summary^a,c^** | | | | | | | | | | |
| --- | --- | --- | --- | --- | --- | --- | --- | --- | --- | --- |
| Model | R | R Square | Adjusted R Square | Std. Error of the Estimate | Change Statistics | | | | | Durbin-Watson |
|  |  |  |  |  | R Square Change | F Change | df1 | df2 | Sig. F Change |  |
|  | .577^b^ | .333 | .241 | 1.75828 | .098 | 4.280 | 1 | 29 | .048 | 1.798 |
| a. OC+ | | | | | | | | | | |
| b. Predictors: (Constant), D_DURAT, dis-ons, LDLc, Tg/HDL | | | | | | | | | | |
| c. Dependent Variable: MSSS | | | | | | | | | | |

| **Coefficients^a,b^** | | | | | | | | | | | |
| --- | --- | --- | --- | --- | --- | --- | --- | --- | --- | --- | --- |
| Model | | Unstandardized Coefficients | | Standardized Coefficients | t | Sig. | Correlations | | | Collinearity Statistics | |
|  |  | B | Std. Error | Beta |  |  | Zero-order | Partial | Part | Tolerance | VIF |
|  | (Constant) | -.687 | 2.009 |  | -.342 | .735 |  |  |  |  |  |
|  | dis-ons | .025 | .056 | .074 | .450 | .656 | .077 | .083 | .068 | .839 | 1.191 |
|  | D_DURAT | -.120 | .070 | -.276 | -1.706 | .099 | -.248 | -.302 | -.259 | .878 | 1.139 |
|  | LDLc | .022 | .008 | .416 | 2.715 | .011 | .378 | .450 | .412 | .979 | 1.021 |
|  | Tg/HDL | .389 | .188 | .326 | 2.069 | .048 | .313 | .359 | .314 | .928 | 1.077 |
| a. OC+ | | | | | | | | | | | |
| b. Dependent Variable: MSSS | | | | | | | | | | | |

| **Residuals Statistics** | | | | | |
| --- | --- | --- | --- | --- | --- |
|  | Minimum | Maximum | Mean | Std. Deviation |  |
| Predicted Value | .9029 | 5.8579 | 2.8036 | 1.15210 |  |
| Residual | -3.08093 | 4.12931 | -.12673 | 1.63972 |  |
| Std. Predicted Value | -1.646 | 2.605 | -.015 | .988 |  |
| Std. Residual | -1.752 | 2.348 | -.072 | .933 |  |

OC-

| **Model Summary^a,c^** | | | | | | | | | | |
| --- | --- | --- | --- | --- | --- | --- | --- | --- | --- | --- |
| Model | R | R Square | Adjusted R Square | Std. Error of the Estimate | Change Statistics | | | | | Durbin-Watson |
|  |  |  |  |  | R Square Change | F Change | df1 | df2 | Sig. F Change |  |
|  | .547^b^ | .299 | .238 | 2.08026 | .076 | 4.971 | 1 | 46 | .031 | 1.168 |
| a. OC- | | | | | | | | | | |
| b. Predictors: (Constant), D_DURAT, dis-ons, AGE, ApoE | | | | | | | | | | |
| c. Dependent Variable: MSSS | | | | | | | | | | |

| **Coefficients^a,b^** | | | | | | | | | | | |
| --- | --- | --- | --- | --- | --- | --- | --- | --- | --- | --- | --- |
| Model | | Unstandardized Coefficients | | Standardized Coefficients | t | Sig. | Correlations | | | Collinearity Statistics | |
|  |  | B | Std. Error | Beta |  |  | Zero-order | Partial | Part | Tolerance | VIF |
|  | (Constant) | -.528 | 1.535 |  | -.344 | .733 |  |  |  |  |  |
|  | AGE | .258 | .216 | .918 | 1.198 | .237 | .240 | .174 | .148 | .026 | 38.497 |
|  | dis-ons | -.154 | .211 | -.520 | -.730 | .469 | .420 | -.107 | -.090 | .030 | 33.242 |
|  | D_DURAT | -.310 | .226 | -.678 | -1.375 | .176 | -.261 | -.199 | -.170 | .063 | 15.954 |
|  | ApoE | .024 | .011 | .296 | 2.229 | .031 | .404 | .312 | .275 | .863 | 1.159 |
| a. OC- | | | | | | | | | | | |
| b. Dependent Variable: MSSS | | | | | | | | | | | |

| **Residuals Statistics** | | | | | |
| --- | --- | --- | --- | --- | --- |
|  | Minimum | Maximum | Mean | Std. Deviation |  |
| Predicted Value | .6470 | 7.1050 | 4.3375 | 1.29056 |  |
| Residual | -5.11941 | 4.50225 | -.02727 | 1.98188 |  |
| Std. Predicted Value | -2.872 | 2.082 | -.041 | .990 |  |
| Std. Residual | -2.461 | 2.164 | -.013 | .953 |  |
